# Elucidating Unique Axonal Dysfunction Between Nitrous Oxide Abuse and Vitamin B12 Deficiency

**DOI:** 10.3389/fneur.2019.00704

**Published:** 2019-07-09

**Authors:** Jowy Tani, Hsing-Yu Weng, Hung-Ju Chen, Tsui-San Chang, Jia-Ying Sung, Cindy Shin-Yi Lin

**Affiliations:** ^1^Department of Neurology, Wan Fang Hospital, Taipei Medical University, Taipei, Taiwan; ^2^Ph.D. Program for Neural Regenerative Medicine, College of Medical Science and Technology, Taipei Medical University and National Health Research Institutes, Taipei, Taiwan; ^3^Taipei Neuroscience Institute, Taipei Medical University, Taipei, Taiwan; ^4^Department of Neurology, School of Medicine, College of Medicine, Taipei Medical University, Taipei, Taiwan; ^5^Graduate Institute of Neural Regenerative Medicine, College of Medical Science and Technology, Taipei Medical University, Taipei, Taiwan; ^6^Faculty of Medicine and Health, Brain and Mind Centre, Central Clinical School, The University of Sydney, Sydney, NSW, Australia

**Keywords:** nerve excitability test, inhalant, nitrous oxide, vitamin B12, myeloneuropathy

## Abstract

**Introduction:** Abuse of nitrous oxide (N_2_O) has an unusually high lifetime prevalence in developed countries and represents a serious concern worldwide. Myeloneuropathy following the inhalant abuse is commonly attributed to the disturbance of vitamin B12 metabolism, with severe motor deficits are often noted. The present study aims to elucidate its underlying pathophysiology.

**Methods:** Eighteen patients with N_2_O abuse or vitamin B12 deficiency were recruited. Comprehensive central and peripheral neuro-diagnostic tests were performed, including whole spine MRI, and thermal quantitative sensory testing (QST). Specifically, paired motor and sensory nerve excitability tests were performed in order to obtain a complete picture of the sensorimotor axonal damage.

**Results:** The mean duration of N_2_O exposure for the N_2_O abuse patients was 17.13 ± 7.23 months. MRI revealed T2 hyperintensity in 87.5% of the N_2_O abuse patients and 50% of the vitamin B12 deficiency patients. In N_2_O abuse patients, the motor nerve excitability test showed decreased in peak response (7.08 ± 0.87 mV, *P* = 0.05), increased latency (7.09 ± 0.28 ms, *P* < 0.01), increased superexcitability (−32.95 ± 1.74%, *P* < 0.05), and decreased accommodation to depolarizing current [TEd (40–60 ms) 56.53 ± 0.70%, *P* < 0.05]; the sensory test showed only decreased peak response (30.54 ± 5.98 μV, *P* < 0.05). Meanwhile, motor test in vitamin B12 deficiency patients showed only decreased accommodation to depolarizing current [TEd (40–60 ms) 55.72 ± 1.60%, *P* < 0.01]; the sensory test showed decreased peak response (25.86 ± 3.44 μV, *P* < 0.05) increased superexcitability (−28.58 ± 3.71%, *P* < 0.001), increased subexcitability (8.31 ± 1.64%, *P* < 0.05), and decreased accommodation to depolarizing current [TEd (peak) 67.31 ± 3.35%, *P* < 0.001].

**Conclusion:** Compared to vitamin B12 deficiency, N_2_O abuse patients showed prominent motor superexcitability changes and less prominent sensory superexcitability changes, hinting a unique pathological process different from that of vitamin B12 deficiency. N_2_O abuse might cause axonal dysfunction not only by blocking methionine metabolism but also by toxicity affecting the paranodal region.

## Introduction

Recreational use of nitrous oxide (N_2_O) is escalating in many countries, including the United States, the United Kingdom, and Taiwan (1, 2). With an unusually high lifetime prevalence in developed countries (38.6% in the UK and 29.4% in the US), N_2_O consistently ranked the seventh most popular drug in the world in the Global Drug Survey (GDS) from 2016 to 2018 (3–5). Abuse of N_2_O frequently leads to overexposure, and subsequently causing myeloneuropathy, subacute combined degeneration, psychosis, megaloblastic bone marrow changes, and pernicious anemia (6). As N_2_O can inactivate methionine synthase that converts homocysteine to methionine via a methylation process, many medical consequences associated with N_2_O overexposure, including peripheral neuropathy, are often attributed to vitamin B12 deficiency (7–9).

Although myeloneuropathy following N_2_O abuse and vitamin B12 deficiency can both result in motor and sensory symptoms, experienced clinicians have often noted somewhat different clinical presentations between the two conditions, where N_2_O abuse might cause severe motor deficits, especially in the lower limbs (10, 11). Johnson et al. have reported such a typical case of nitrous oxide abuse leading to significant distal limbs weakness, a clinical feature less likely to be observed in vitamin B12 deficiency from other causes (12).

Certain reports have suggested that N_2_O neurotoxicity might cause neural injury on top of vitamin B12 deficiency, but the exact pathophysiology of N_2_O-induced myeloneuropathy is still unclear (6, 13). The present study is the first that uses comprehensive neurodiagnostic tests covering central and peripheral nervous systems, including whole spine MRI, conventional nerve conduction study (NCS), thermal quantitative sensory test (QST), and the nerve excitability tests to study the myeloneuropathy. In particular, the nerve excitability test has been shown to be able to provide valuable data on the nodal, paranodal, and internodal regions of axons in various peripheral nerve diseases (14, 15), and reveal changes in axonal properties that cannot be detected using conventional NCS. It is hoped that paired motor and sensory nerve excitability test utilized in the study would provide a complete picture of the sensorimotor axonal damage, and further elucidate the pathophysiology underlying the inhalant abuse that could cause catastrophic consequences.

## Methods

A total of 18 patients with either N_2_O abuse or vitamin B12 deficiency due to other causes were recruited for the study. Each patient received clinical evaluation (including history taking, complete physical examination, and neurologic examination); laboratory examinations for levels of vitamin B12, folic acid, and homocysteine; conventional NCS; and paired motor and sensory nerve excitability tests; certain patients received a thermal QST. All patients received whole spine MRI, except one vitamin B12 deficiency patient.

All N_2_O abuse patients had multiple exposures to N_2_O inhalation (1). On the other hand, a patient was considered to have vitamin B12 deficiency if the measured serum vitamin B12 level was <150 pg/ml (9, 16–18). Patients with carpal tunnel syndrome, hyperkalemia/hypokalemia, or with other potential causes for sensory polyneuropathy such as diabetes mellitus, alcohol abuse, and uremia were excluded based on clinical assessments and NCS results. All patients enrolled in the study were recruited from Wan Fang Hospital, Taipei Medical University, Taipei, Taiwan. Control nerve excitability data were obtained from 39 healthy control (HC) subjects that were divided into two age cohorts, HC1 with a mean age of 22.67 ± 0.21 years (*n* = 6) and HC2 with a mean age of 62.11 ± 7.51 years (*n* = 33). HC1 was age-matched to the N_2_O abuse patients, and HC2 was age-matched to the vitamin B12 deficiency patients.

This study was carried out in accordance with the recommendations of the Joint Institutional Review Board of Taipei Medical University. The protocol was approved by the Joint Institutional Review Board of Taipei Medical University. All subjects gave written informed consent in accordance with the Declaration of Helsinki.

### Clinical Evaluation, NCS, and QST

The Medical Research Council (MRC) strength score combined for 12 specified muscle groups (the MRC sum score; ranging from 0 [paralysis] to 60 [normal strength]) (19) and Neuropathy Impairment Score in the Lower Limbs (NIS-LL; ranging from 0 [normal] to 88 [total impairment]) (20) were obtained during neurological examination. Conventional NCS assessing the median, ulnar, peroneal, tibial, and sural nerves were performed in all subjects using standard clinical neurophysiology equipment. Cold and warm thermal QSTs were performed in the upper and lower limbs in certain patients.

### Nerve Excitability Testing

Nerve excitability studies were performed by stimulating the nerve median at the wrist according to previously described protocols, with skin temperature over the wrist maintained at a minimum of 32.0°C (14, 15). Paired recordings of the motor and sensory nerve excitability indices were obtained for each subject. Compound muscle action potentials (CMAPs) were recorded from the abductor pollicis brevis muscle while sensory nerve action potentials (SNAPs) were recorded from the index finger, Stimulation and recording were manipulated by software (QTRAC version 8/11/2014; Institute of Neurology, London, U.K.), and stimulus current was administered using an isolated linear bipolar constant-current stimulator (DS5; Digitimer, Welwyn Garden City, U.K.). The changes in current required to produce a target potential corresponding to predetermined target CMAP or SNAP (e.g., 40% of maximum) were tracked. Latency was defined as the time delay (ms) between stimulus onset and the peak of the CMAP or SNAP response. Stimulus threshold was defined as the current (mA) that is required to produce amplitudes of CMAP or SNAP response of the target amplitude.

The nerve excitability protocol incorporated the following recordings: (1) a stimulus-response (SR) curve; (2) strength-duration (SD) relationship, which determined strength-duration time constant (SDTC); (3) threshold electrotonus (TE) utilizing subthreshold 100-ms polarizing currents in both depolarizing (TEd; +40%) and hyperpolarizing (TEh; −40%) directions to change the potential difference across the internodal membrane; and (4) recovery cycle (RC) using a paired-pulse paradigm with a supramaximal conditioning stimulus followed by a test stimulus at interstimulus intervals from 2 to 200 ms. Superexcitability was measured as the maximal threshold reduction and subexcitability as the maximal threshold increase after an interstimulus interval of 10 ms.

### Statistical Analysis

Nerve excitability recording data of N_2_O abuse patients, vitamin B12 deficiency patients, and healthy controls were analyzed by using unpaired *T*-tests or Mann-Whitney U-tests, depending on normality. Equality of variances was calculated with Levene's test. Correlation studies were performed with Pearson's R. Data analysis was performed using Statistical Package for the Social Sciences (SPSS) for Windows version 21 (SPSS Inc., Chicago, U.S.A.) or QTRAC software. All data are presented as the mean ± standard error of the mean. *P*-values were considered significant if ≤ 0.05.

## Results

### Patient Clinical Profiles

**Figure 1:**
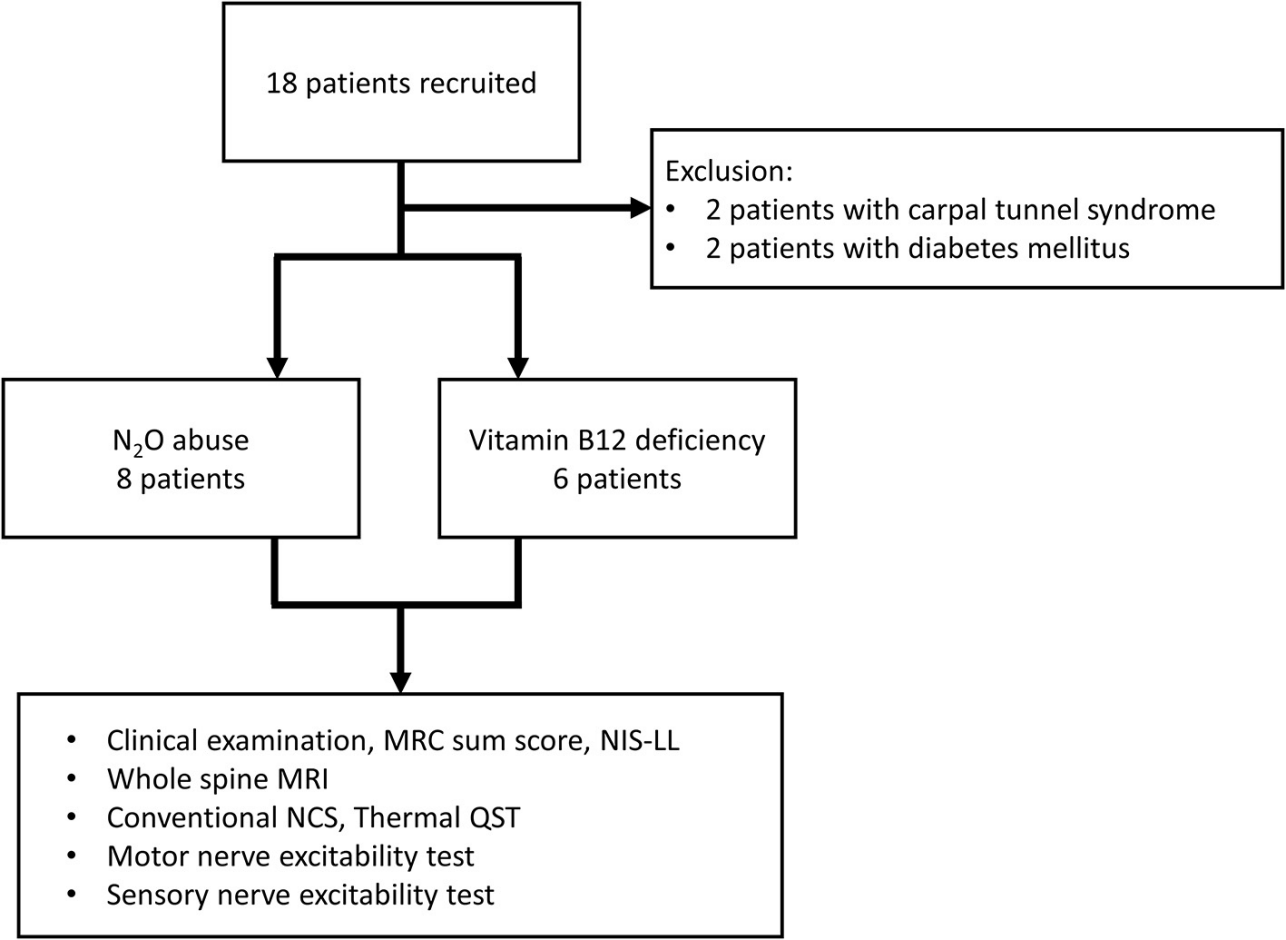
This flowchart depicts the recruitment and the subjects involved in the final data analysis. Eighteen patients were categorized into the N_2_O abuse group and vitamin B12 deficiency group. MRC, Medical Research Council; NIS-LL, Neuropathy Impairment Score in the Lower Limbs; MRI, magnetic resonance imaging; NCS, nerve conduction study; QST, quantitative sensory test.

Eighteen patients were recruited and categorized into N_2_O abuse patients (*n* = 8) and vitamin B12 deficiency patients (*n* = 6). Two patients were excluded due to comorbid diabetes, and two for carpal tunnel syndrome (Figure 1). The clinical, MRI, and laboratory profiles for the patients were listed in Table 1. The average duration of N_2_O exposure for the N_2_O abuse group was 17.13 ± 7.23 months. The MRC sum score was 51.50 ± 2.87 for the N_2_O abuse group, and 59.67 ± 0.33 for the vitamin B12 deficiency group; NIS-LL was 27.00 ± 5.83 for the N_2_O abuse group, and 10.00 ± 4.48 for the vitamin B12 deficiency group.

**Table 1:** Patients clinical, MRI, and laboratory profiles.

	**N_**2**_O abuse**	**Vitamin B12 deficiency**
Sex: male/female (number)	5/3	4/2
Age (year)	26.75 ± 2.59	60.5 ± 8.74
Duration of N_2_O abuse (months)	17.13 ± 7.23	–
MRC sum score	51.50 ± 2.87	59.67 ± 0.33
NIS-LL	27.00 ± 5.83	10.00 ± 4.47
Vitamin B12 level (pg/ml)	236.33 ± 52.80	111.50 ± 15.46^*^
Homocysteine level (μmol/l)	44.84 ± 12.00^*^	51.68 ± 25.52^*^
**UPPER LIMBS CLINICAL FINDINGS**		
Weakness (%)	37.50	0
Upper limbs MRC sum score	27.50 ± 1.55	30.00 ± 0.00
Abnormal pain/temperature sensation (%)	75.00	16.67
Abnormal vibratory sensation/proprioception (%)	37.50	0
Hypo/hyperreflexia (%)	62.50	0
Ataxia (%)	0	0
**LOWER LIMBS CLINICAL FINDINGS**		
Weakness (%)	75.00	33.33
Lower limbs MRC sum score	24.00 ± 2.20	29.67 ± 0.33
Abnormal pain/temperature sensation (%)	100.00	66.67
Abnormal vibratory sensation/proprioception (%)	50.00	66.67
Hypo/hyperreflexia (%)	75.00	50.00
Ataxia (%)	12.50	0
Autonomic dysfunction (%)	25.00	33.33
**MRI FINDING**		
T2 hyperintensity on whole spine MRI (%)	87.50	50.00
**MOTOR CONDUCTION VELOCITY**		
Median nerve (m/s)	47.19 ± 2.83^*^	50.59 ± 2.58
Ulnar nerve (m/s)	46.31 ± 4.31^*^	52.50 ± 2.80
Tibial nerve (m/s)	17.00 ± 6.46^*^	33.33 ± 6.74^*^
Peroneal nerve (m/s)	15.69 ± 6.71^*^	34.25 ± 6.90^*^
**ABNORMAL MOTOR CONDUCTION VELOCITY**		
Median nerve (%)	87.50	40.00
Ulnar nerve (%)	87.50	100.00
Tibial nerve (%)	62.50	20.00
Peroneal nerve (%)	62.50	20.00
**SENSORY CONDUCTION VELOCITY**		
Median nerve (m/s)	47.38 ± 2.64^*^	51.92 ± 5.75
Ulnar nerve (m/s)	49.13 ± 2.09^*^	51.11 ± 4.69
Sural nerve (m/s)	24.06 ± 9.27^*^	35.17 ± 11.34^*^
**ABNORMAL SENSORY CONDUCTION VELOCITY**		
Median nerve (%)	75.00	40.00
Ulnar nerve (%)	50.00	80.00
Sural nerve (%)	50.00	40.00
**THERMAL QST**		
Upper limb warm threshold (*Z*-score)	4.05 ± 1.36^*^	2.95 ± 0.65^*^
Upper limb cold threshold (*Z*-score)	−2.00 ± 0.61^*^	−0.38 ± 0.80
Lower limb warm threshold (*Z*-score)	1.77 ± 0.93	3.10 ± 0.03^*^
Lower limb cold threshold (*Z*-score)	−1.76 ± 0.86	−4.21 ± 1.05^*^

*The reported values of patient profiles, laboratory data, and thermal QST data represent the mean ± standard error. The reported values of abnormal nerve conduction velocity data, clinical findings, and MRI profile represent percentage*.

*MRC, Medical Research Council; NIS-LL, Neuropathy Impairment Score in the Lower Limbs; QST, Quantitative sensory test; MRI, magnetic resonance imaging*;

* *mean data are out of the normal range for this laboratory*.

The vitamin B12 level was 236.33 ± 52.80 pg/ml in the vitamin B12 deficiency group and 111.50 ± 15.46 pg/ml in the N_2_O abuse group. The homocysteine level was elevated in both the N_2_O abuse group (44.84 ± 12.00 μmol/l) and vitamin B12 deficiency group (51.68 ± 25.52 pg/ml).

A summary of the patients' neurological signs and symptoms are shown in Table 1. Weakness was more prevalent in the N_2_O abuse group than in the vitamin B12 group, both in the upper (37.50 vs. 0.00%) and lower (75.00 vs. 33.33%) limbs. While sensory abnormalities are more prevalent in N_2_O abuse group than in the vitamin B12 group in the upper limbs, the prevalence of sensory abnormalities of both conditions are similar in the lower limbs.

**Figure 2:**
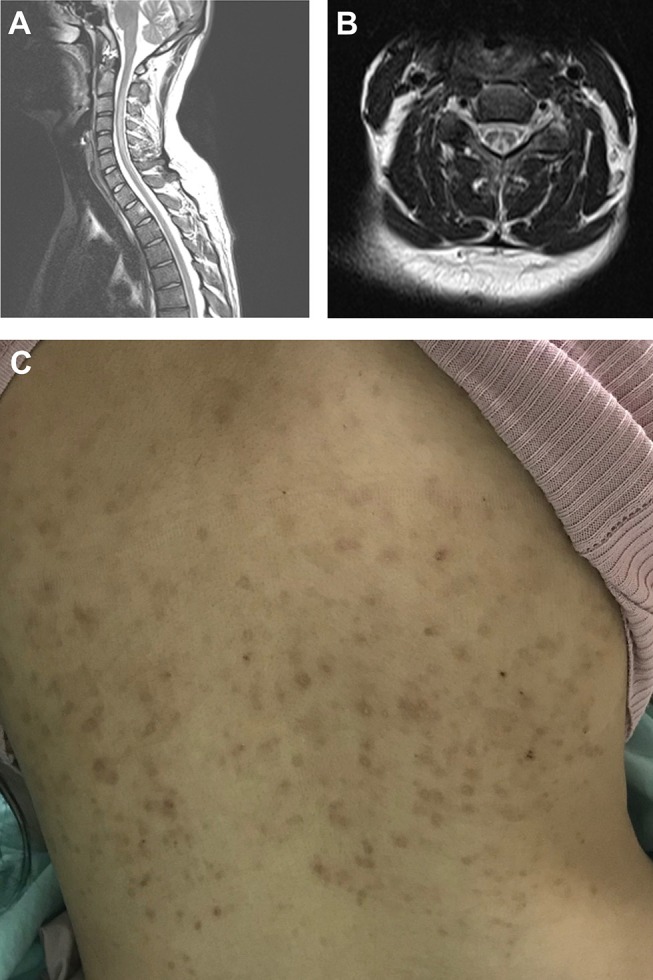
Typical MRI showing cervical spine T2 hyperintense lesion in an N+O abuse patient. **(A)** Sagittal view and **(B)** axial view. **(C)** Hyperpigmented maculopapular rash observed in an N_2_O abuse patient.

The whole spine MRI revealed T2 hyperintense lesions in the posterior columns in 87.5% of the N_2_O abuse patients (100% were cervical spine lesions), and in 50% of the vitamin B12 deficiency patients (66.6% were cervical spine lesions and 33.3% were thoracolumbar lesions). Figures 2A,B shows MRI of cervical cord hyperintense changes in T2-weighted images from a case with N_2_O abuse. Furthermore, Figure 2C showed a hyperpigmented maculopapular rash, a feature that could be observed in N_2_O abuse patients, on one of the patients in the nitrous oxide abuse group.

NCS revealed motor and sensory slowing in conduction velocity in both N_2_O abuse and vitamin B12 deficiency patients. Thermal QST in the upper limb (N_2_O abuse warm threshold *Z*-score: 4.05 ± 1.36, cold threshold *Z*-score: −2.00 ± 0.61; Vitamin B12 deficiency warm threshold *Z*-score: 2.95 ± 0.65, cold threshold *Z*-score: −0.38 ± 0.80) and lower limb (N_2_O abuse warm threshold *Z*-score: 1.77 ± 0.93, cold threshold *Z*-score: −1.76 ± 0.86; Vitamin B12 deficiency warm threshold *Z*-score: 3.10 ± 0.03, cold threshold *Z*-score: −4.21 ± 1.05) confirmed abnormal temperature sensation in the patients.

### Motor Axonal Dysfunction in N_2_O Abuse and Vitamin B12 Deficiency Patients

**Figure 3:**
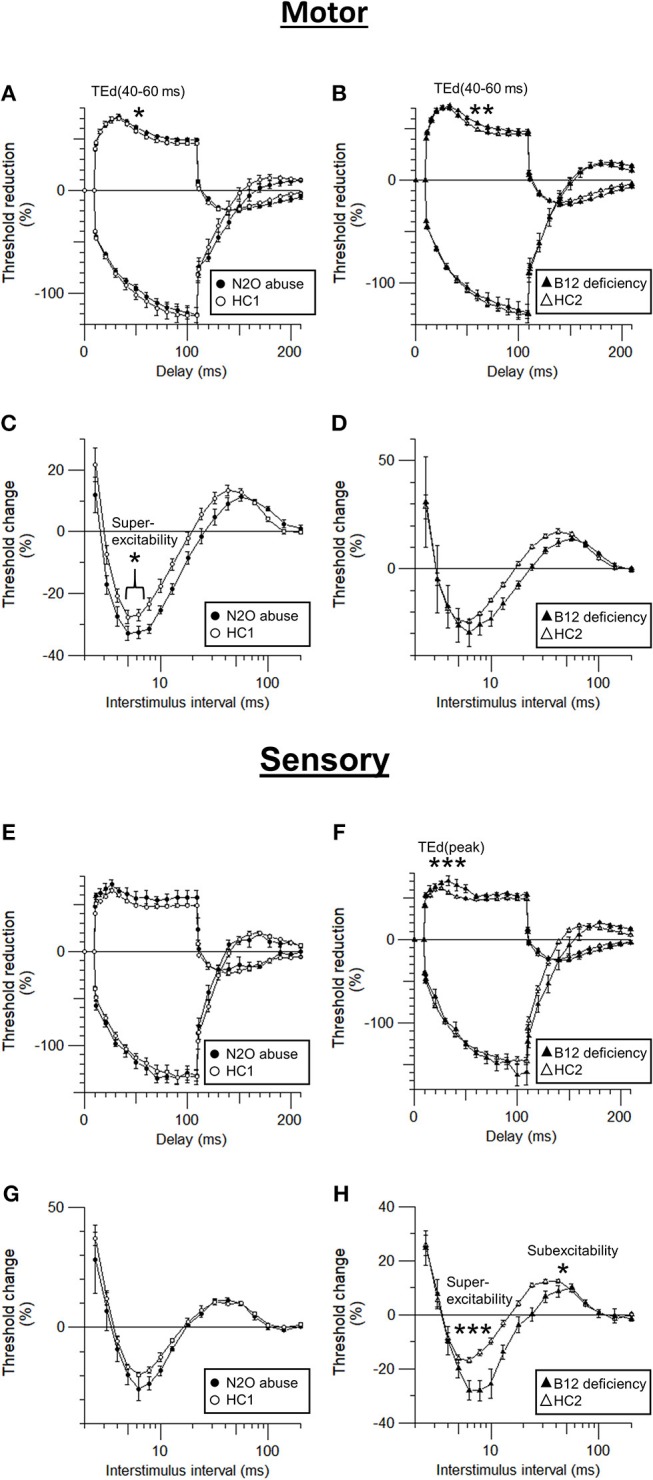
Comparison of sensory and motor axonal dysfunction parameters in N_2_O abuse and vitamin B12 deficiency patients. **(A,B)** motor threshold electrotonus, **(C,D)** motor recovery cycle, **(E,F)** sensory threshold electrotonus, and **(G,H)** sensory recovery cycle. *p < 0.05, **p < 0.01, ***p < 0.001.

A comparison of the motor and sensory nerve excitability parameters between groups are shown in Table 2. Figure 3 shows the motor and sensory recovery cycles and threshold electrotonus in N_2_O abuse and vitamin B12 deficiency patients.

**Table 2:** Comparison of sensory and motor nerve excitability parameters between groups.

**Axonal properties**	**N**_****2****_**O abuse vs. age-matched controls**			**B12 deficient vs. age-matched controls**		
	**N_**2**_O abuse**	**HC1**	***p*-value**	**B12 def**.	**HC2**	***p*-value**
**MOTOR PARAMETERS**						
Stimulus for 50% CMAP (mA)	3.58 ± 0.79	2.46 ± 0.17	NS	4.59 ± 0.73	2.76 ± 0.14	*p* < 0.01
Peak response (mV)	7.08 ± 0.87	9.7 ± 0.64	*p* = 0.05	8.14 ± 1.21	8.31 ± 0.46	NS
Latency (ms)	7.09 ± 0.28	5.59 ± 0.15	*p* < 0.01	7.11 ± 0.45	6.62 ± 0.17	NS
Motor SDTC (ms)	0.52 ± 0.04	0.49 ± 0.04	NS	0.48 ± 0.03	0.46 ± 0.01	NS
RRP (ms)	3.00 ± 0.17	2.99 ± 0.10	NS	4.14 ± 0.54	3.12 ± 0.09	NS
Superexcitability (%)	−32.95 ± 1.74	−25.98 ± 2.07	*p* < 0.05	−27.19 ± 5.71	−23.98 ± 0.98	NS
Subexcitability (%)	11.19 ± 1.01	12.63 ± 1.46	NS	12.83 ± 1.32	16.44 ± 0.94	NS
TEd (40–60 ms) (%)	56.53 ± 0.70	52.39 ± 1.77	*p* < 0.05	55.72 ± 1.60	50.04 ± 0.78	*p* < 0.01
TEd (peak) (%)	68.97 ± 2.30	68.12 ± 1.67	NS	70.58 ± 1.34	68.33 ± 0.69	NS
TEh (90–100 ms) (%)	−119.86 ± 6.84	−121.92 ± 8.00	NS	−127.76 ± 8.91	−129.87 ± 3.82	NS
**SENSORY PARAMETERS**						
Stimulus for 50% SNAP (mA)	3.28 ± 0.60	1.96 ± 0.13	*p* < 0.05	3.65 ± 0.78	2.24 ± 0.18	*p* < 0.05
Peak response (μV)	30.54 ± 5.98	55.48 ± 8.87	*p* < 0.05	25.86 ± 3.44	39.36 ± 2.47	*p* < 0.05
Latency (ms)	3.57 ± 0.30	2.9 ± 0.10	NS	3.45 ± 0.23	3.33 ± 0.06	NS
Sensory SDTC (ms)	2.58 ± 0.34	0.57 ± 0.03	NS	0.50 ± 0.03	0.63 ± 0.02	*p* < 0.01
RRP (ms)	3.52 ± 0.33	3.71 ± 0.10	NS	3.57 ± 0.26	3.44 ± 0.12	NS
Superexcitability (%)	−23.65 ± 3.50	−18.07 ± 1.03	NS	−28.58 ± 3.71	−16.61 ± 1.06	*p* < 0.001
Subexcitability (%)	10.88 ± 0.74	10.46 ± 0.71	NS	8.31 ± 1.64	12.39 ± 0.61	*p* < 0.05
TEd (40–60 ms) (%)	56.43 ± 6.25	47.98 ± 1.64	NS	54.60 ± 1.26	48.53 ± 0.62	*p* < 0.001
TEd (peak) (%)	68.56 ± 5.52	60.62 ± 1.03	NS	67.31 ± 3.35	59.36 ± 0.51	*p* < 0.001
TEh (90–100 ms) (%)	−130.08 ± 5.73	−133.05 ± 7.68	NS	−161.35 ± 13.79	−145.44 ± 4.35	NS

*HC, healthy control; CMAP, compound muscle action potential; SNAP, sensory nerve action potential; SDTC, estrength-duration time constant; RRP, relative refractory period; NS, not statistically significant. The reported values represent the mean ± standard error and the p-value from t-tests or Mann-Whitney U-tests of patient groups vs. age-matched healthy controls, depending on normality*.

Compared to those of HC, the motor nerve excitability of the patients with N_2_O abuse showed a trend of decreased peak response (7.08 ± 0.87 mV, *p* = 0.05), increased latency (7.09 ± 0.28 ms, *p* < 0.01), increased superexcitability (−32.95 ± 1.74%, *p* < 0.05), and decreased TE accommodation to depolarizing current [TEd [40–60 ms] 56.53 ± 0.70%, *p* < 0.05].

On the other hand, the motor nerve excitability test of vitamin B12 deficiency patients showed only increased stimulus for 50% CMAP (4.59 ± 0.73 mA, *p* < 0.01) and decreased TE accommodation toward depolarization in TEd (40–60 ms) (55.72 ±1.60%, *p* < 0.01).

More prominent motor superexcitability changes in N_2_O abuse patients compared to those seen in vitamin B12 deficiency patients clearly suggested motor axonal dysfunction is more severe in N_2_O abuse, possibly affecting the paranodal region.

### Sensory Axonal Dysfunction in N_2_O Abuse and Vitamin B12 Deficiency Patients

The sensory nerve excitability test of the patients with N_2_O abuse showed increased stimulus for 50% SNAP (3.28 ± 0.60 mA, *p* < 0.05) and decreased peak response (30.54 ± 5.98 μV, *p* < 0.05).

Sensory nerve excitability test of the vitamin B12 deficiency patients also showed increased stimulus for 50% SNAP (3.65 ± 0.78 mA, *p* < 0.05) and decreased peak response (25.86 ± 3.44 μV, *p* < 0.05). Moreover, it revealed additional evidence of sensory axonal dysfunction including decreased SDTC (0.50 ± 0.03, *p* < 0.01), increased superexcitability (−28.58 ± 3.71%, *p* < 0.001), decreased subexcitability (8.31 ± 1.64%, *p* < 0.05), and decreased TE accommodation toward depolarizing current (TEd[peak] 67.31 ± 3.35, *p* < 0.001).

The fact that superexcitability and other TE & RC parameters showed significant changes in vitamin B12 deficiency but not in N_2_O abuse suggested that vitamin B12 deficiency patients suffered from more severe sensory axonal dysfunction, compared to N_2_O abuse patients.

Correlational study between peak response and latency for motor (*R* = −0.65, *p* = 0.08) and sensory (*R* = −0.69, *p* = 0.06) axon in N_2_O abuse patients, as well as for sensory axon in vitamin B12 deficiency patients (*R* = −0.11, *p* = 0.80), suggested that decrease in peak response observed in the present study might be related to axonal loss instead of temporal dispersion.

### Correlation Studies Between Clinical Parameters and Excitability Parameters

In order to clarify the cumulative effect of prolonged intermittent exposure of N_2_O on the axon, the present study performed correlation analysis between the duration of intermittent N_2_O exposure and motor and sensory excitability parameters in the N_2_O abuse group. The analysis demonstrated that the duration of intermittent N_2_O exposure is significantly correlated to both motor and sensory excitability parameters, specifically N_2_O exposure is correlated to subexcitability (*R* = 0.85, *p* < 0.01) for motor and TEd (peak) (*R* = 0.76, *p* < 0.05) for sensory axons.

Also, vitamin B12 level was correlated with stimulus for 50% SNAP response (*R* = −0.81, *p* < 0.05) and sensory rheobase (*R* = −0.90, *p* < 0.05) of vitamin B12 deficiency patients. Interestingly, MRC strength score of tested muscle is correlated with TEh(overshoot) (*R* = −0.82, p < 0.05) in motor axonal study of N_2_O abuse patients.

In the sensory axonal study of N_2_O abuse patients, the present study found that upper limb warm threshold *Z*-score was correlated with refractoriness at 2.5 ms (*R* = 0.97, *p* < 0.05), superexcitability (*R* = −0.98, *p* < 0.05). Upper limb cold threshold *Z*-score was correlated with superexcitability (*R* = 0.97, *p* < 0.05) and latency (*R* = −0.98, *p* < 0.05). Lower limb warm threshold *Z*-score was correlated with subexcitability (*R* = −0.98, *p* < 0.05).

Also, in the sensory axonal study of vitamin B12 deficiency patients, the present study found that upper limb cold threshold *Z*-score was correlated with peak response (*R* = 0.93, *p* < 0.05) and TEd (40–60 ms) (*R* = 0.89, *p* < 0.05). Lower limb warm threshold *Z*-score was correlated with refractoriness at 2.5 ms (*R* = 0.94, *p* < 0.05). Lower limb cold threshold *Z*-score was correlated with peak response (*R* = 0.95, *p* < 0.05), TEd (10–20 ms) (*R* = 0.9149, *p* < 0.05), TEd (40–60ms) (*R* = 0.91, *p* < 0.05), and TEd (peak) (*R* = 0.96, *p* < 0.05).

## Discussion

**Figure 4:**
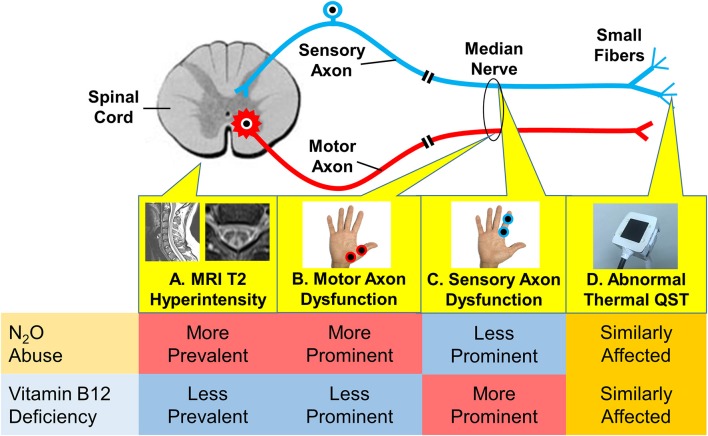
Different patterns of myeloneuropathy in N_2_O abuse and vitamin B12 deficiency, as revealed by different diagnostic modalities in the present study. **(A)** Spinal MRI revealed more prevalent T2 hyperintensity in the N_2_O abuse group. **(B)** The motor axonal study revealed more prominent dysfunction in the N_2_O abuse group. **(C)** The sensory axonal study revealed more prominent dysfunction in the vitamin B12 deficiency group. **(D)** Thermal QST (quantitative sensory testing) revealed that temperature sensation was similarly affected in both groups.

In the present study, the spinal MRI, motor and sensory nerve excitability tests, and thermal QST revealed evidence of myeloneuropathy in both the N_2_O abuse and vitamin B12 deficiency patients. Nevertheless, the pattern of myeloneuropathy appears to be different between the two groups (Figure 4). Thermal QST revealed that temperature sensation could be affected by both N_2_O abuse and vitamin B12 deficiency.

While spinal MRI abnormalities have been previously reported in both vitamin B12 deficiency (21) and N_2_O overexposure (12, 22, 23), we have not found studies that compare spinal MRI abnormalities in the two conditions; the present study revealed that spinal cord T2 hyperintense lesions were more prevalent in the N_2_O abuse group. Nerve excitability test can reveal evidence of axonal dysfunctions that cannot be detected by conventional NCS, allowing us to gain insights into the unique pathophysiology associated with N_2_O abuse.

### A Different Pattern of Axonal Dysfunction in N_2_O Abuse and Vitamin B12 Deficiency

Compared to vitamin B12 deficiency patients, N_2_O abuse patients showed a unique nerve excitability pattern showing prominent motor superexcitability changes and less prominent sensory superexcitability changes. This provides evidence for the underlying pathological mechanism that affects the two conditions might be different, as discussed below.

### Obvious Motor Axonal Dysfunction in N_2_O Abuse

The nerve excitability test in patients with N_2_O abuse showed decreased peak response, increased latency, decreased accommodation toward depolarizing current, and increased superexcitability. The increase in superexcitability may correspond to an alteration in fast K^+^ channels in the axonal paranodal region, while decreased accommodation toward depolarizing current signifies an alteration in nodal and internodal slow K^+^ channels (24). Similar changes in superexcitability and increased TE accommodation toward depolarizing current have been noted in other demyelinating diseases in previous studies. In particular, “fanning-out” of TE and increased superexcitability were seen in demyelinating diseases such as chronic inflammatory demyelinating polyneuropathy and multifocal motor neuropathy and was thought to possibly be related to compensatory Na^+^/K^+^ pump action in measured nerve segments (25–27). Such changes observed in patients with N_2_O abuse may similarly be related to demyelination of proximal axons. The decrease in peak response noted in the patients may signify motor axonal loss.

Patients with vitamin B12 deficiency similarly showed motor axonal dysfunction, particularly, decreased accommodation toward depolarization. Although the changes are still in accordance with demyelination, the motor axonal damage appears to be less prominent than those in our N_2_O abuse patients, as there are no significant changes in the RC. Moreover, no significant reduction in peak response is observed.

The motor axonal dysfunction pattern in both patients with N_2_O abuse and vitamin B12 deficiency is compatible with motor demyelination. However, patients with N_2_O abuse appear to have more prominent dysfunction in motor axons. The finding is in accordance with the clinical findings, in which patients with N_2_O abuse show prominent motor deficits compared to those with vitamin B12 deficiency. Previous reports also described disproportionate motor amplitude reduction in the lower limbs in NCS of N_2_O abuse patients (1, 12).

Traditionally, neuropathy induced by N_2_O abuse was attributed to the effect of N_2_O on the inactivation of vitamin B12 through blockage of methionine synthase, which converts homocysteine to methionine via a methylation process. The blockage could lead to the reduction of the vitamin B12 level, which then leads to hypomyelination and abnormal myelination (9, 28). It would concurrently lead to elevation of the homocysteine level, which might cause inflammation, oxidative stress, and microvascular disease (9, 29–31). Nevertheless, previous reports have also mentioned that N_2_O might exert its neurotoxic effect independent of vitamin B12 deficiency, via mechanisms such as antagonism of N-methyl-D-aspartate (NMDA) receptors (6, 13). Certain reports reported ischemic pathological changes associated with nitrous oxide use (32).

While the exact pathophysiology of N_2_O-induced myeloneuropathy remains poorly understood, as mentioned in the discussion above, the prominent changes in motor superexcitability observed in the present study might be an opportunity to elucidate further whether N_2_O abuse causes unique pathophysiological changes in motor axons, especially in the paranodal region.

### Prominent Sensory Axonal Dysfunction in Vitamin B12 Deficiency Patients

Although the results of the sensory nerve excitability test of patients with N_2_O abuse showed a decreased peak response, they showed only a trend of increased superexcitability and “fanning-out” toward depolarizing and hyperpolarizing current. These axonal changes are less pronounced than those of vitamin B12 deficiency patients, which not only showed peak reduction but also showed obvious signs of demyelination such as decreased accommodation toward depolarizing current, increased superexcitability, and decreased subexcitability. Decreased sensory SDTC in vitamin B12 deficiency patients, signifying alterations in persistent Na channels, is likewise compatible with hyperpolarization.

Sensory axonal dysfunction patterns in both N_2_O abuse and vitamin B12 deficiency patients are similarly compatible with demyelination. Prominent changes in patients with vitamin B12 deficiency reflected significant sensory axonal dysfunction. These findings are in accordance with the clinical situation, in which vitamin B12 deficiency patients have more significant sensory complaints than N_2_O abuse patients.

Previous nerve biopsy reports have shown that vitamin B12 deficiency could cause a combination of axonal degeneration and demyelination in vitamin B12 deficiency (33). A sural nerve biopsy report of N_2_O abuse revealed evidence for both axonal degeneration and demyelination. Specifically, it found prominent evidence for axonal degeneration such as axonal swelling, shrunken axon, and varying degrees of myelin ovoid formation. However, it also presented evidence for focal demyelination and subsequent remyelination, including the occasional focal area of myelin loss, focal sites of denuded myelin, and short internodal segments of myelin (34).

Decreased peak response, together with prominent TE and RC changes seen in motor axonal study of N_2_O abuse patients and sensory axonal study of vitamin B12 deficiency patients probably reflect a combined effect of axonal loss and focal demyelination, in accordance with previous pathology report (33, 34). Prominent motor superexcitability changes in N_2_O abuse patients might be related to toxicity involving paranodal region, possibly affecting fast K^+^ channel.

Less prominent TE and RC changes observed in the sensory axonal study of N_2_O abuse patient and motor axonal study of vitamin B12 deficiency patient probably indicate that focal demyelination and toxicity involving paranodal region has less effect on the sensory axon of N_2_O abuse patients and motor axon of vitamin B12 deficiency patient.

### Potential Future Roles of Nerve Excitability Test in the Diagnosis of Myeloneuropathies

The ability of the nerve excitability test to detect axonal dysfunction in both N_2_O abuse and vitamin B12 deficiency suggests that the tool could play a role in the diagnosis of neuropathy in both conditions. A previous study has described the axonal dysfunction pattern in myelopathy and radiculopathy, and the present study has further elucidated axonal dysfunction pattern in N_2_O abuse and vitamin B12 deficiency myeloneuropathy, further clarifying axonal dysfunction patterns that could be observed when injury affects the spinal cord, root, and/or peripheral nerve (35). The present study has shown that the tool can distinguish motor and sensory nerve injury much more clearly than the NCS, and may pave the way for the nerve excitability test to become another useful neurophysiologic diagnostic tool in the evaluation of myeloneuropathies, alongside with NCS. In particular, for patients presenting with a myeloneuropathy with unclear history, a nerve excitability test result showing the unique motor predominant axonal dysfunction as unveiled by the present study could support the diagnosis of N_2_O abuse.

The fact that motor subexcitability and sensory TEd (peak) are correlated with N_2_O exposure duration, and that motor TEh(overshoot) are correlated with MRC strength score of tested muscle in N_2_O abuse, suggested that nerve excitability parameters may have the potential to monitor axonal damages caused by N_2_O exposure, and may further add to the value of the nerve excitability test as an evaluation tool for the myeloneuropathy. Correlation between vitamin B12 level with numerous sensory axonal study parameters suggested that the parameters reflect axonal dysfunction related to vitamin B12 deficiency. Correlational study results also showed that RC and TE parameters are correlated with sensory perception threshold in the patients, but the interpretation of the results should be very careful, as the nerve excitability test was not specifically designed to evaluate small fiber function.

In summary, the present study has revealed that the nerve excitability test could detect motor and sensory axonal dysfunction in both N_2_O abuse and vitamin B12 deficient patients. Follow-up studies with more cases would be beneficial to confirm the unique axonal dysfunction pattern in N_2_O abuse and to explore treatment strategies for the abuse of the inhalant based on its axonal pathophysiology.

## Data Availability

The raw data supporting the conclusions of this manuscript will be made available by the authors, without undue reservation, to any qualified researcher.

## Ethics Statement

This study was carried out in accordance with the recommendations of the Joint Institutional Review Board, Taipei Medical University with written informed consent from all subjects. All subjects gave written informed consent in accordance with the Declaration of Helsinki. The protocol was approved by the Joint Institutional Review Board, Taipei Medical University.

## Author Contributions

JT and J-YS contributed to the study design. JT, H-JC, T-SC, H-YW, and J-YS contributed to the data collection. JT, T-SC, J-YS, and CL contributed to the data analysis and interpretation. JT, J-YS, and CL contributed to the manuscript preparation. All authors approved the final version of the manuscript.

### Conflict of Interest Statement

The authors declare that the research was conducted in the absence of any commercial or financial relationships that could be construed as a potential conflict of interest.
